# THE ASSOCIATION OF ANXIETY WITH INDOOR AND OUTDOOR FALLS AMONG COMMUNITY-DWELLING OLDER ADULTS

**DOI:** 10.1093/geroni/igad104.0782

**Published:** 2023-12-21

**Authors:** Lingming Chen, Elizabeth Procter-Gray, Linda Churchill, Meng Zhang, Qun Le, Sarah Berry, Marian Hannan, Wenjun Li

**Affiliations:** University of Massachusetts Lowell, Lowell, Massachusetts, United States; University of Massachusetts Lowell, Lowell, Massachusetts, United States; University of Massachusetts, Lowell, Lowell, Massachusetts, United States; University of Massachusetts Lowell, Lowell, Massachusetts, United States; University of Massachusetts Lowell, Lowell, Massachusetts, United States; Hebrew Rehabilitation Center, Boston, Massachusetts, United States; Harvard Medical School, Boston, Massachusetts, United States; University of Massachusetts Lowell, Lowell, Massachusetts, United States

## Abstract

One in three older adults aged 65 years or greater fall at least once each year. The effects of psychosocial factors on falls remain to be fully understood. This study examined the association of anxiety with subsequent indoor and outdoor falls among older adults. The prospective cohort study enrolled 359 community-dwelling older adults aged 65-95 years old in central Massachusetts (2018-2020). Anxiety at baseline was measured by the Beck Anxiety Inventory. Falls were reported on monthly falls calendars, and when a fall was reported, the circumstances were collected via telephone interview. Negative binomial models were used to estimate the effect of anxiety on indoor and outdoor falls, separately. Models were adjusting for sociodemographic, physical health (BMI, bodily pain, comorbidities), depression, stress, functional status (Activities of Daily Living, chair standing test), physical activity, drinking, smoking, the fear of falling and ever fallen in the past year. Overall, the rates of indoor falls and outdoor falls were 41 per 100 person-years and 46 per 100 person-years, respectively. Anxiety was a statistically significant predictor of indoor falls; for every one point increased in Beck Anxiety Inventory, there was a 6% increase in the incident rate of indoor falls after adjusting for covariables listed above (IRR (95% CI): 1.06 (1.01-1.11)). Anxiety was not significantly associated with outdoor falls (IRR (95% CI): 0.98 (0.93-1.02)). In conclusion, anxiety was associated with higher rate of indoor falls but not outdoor falls. Future falls prevention should consider the differential impact of anxiety on indoor and outdoor falls.

